# Realistic biomarkers from plasma extracellular vesicles for detection of beryllium exposure

**DOI:** 10.1007/s00420-022-01871-7

**Published:** 2022-05-12

**Authors:** Raju S. R. Adduri, Ravikiran Vasireddy, Margaret M. Mroz, Anisha Bhakta, Yang Li, Zhe Chen, Jeffrey W. Miller, Karen Y. Velasco-Alzate, Vanathi Gopalakrishnan, Lisa A. Maier, Li Li, Nagarjun V. Konduru

**Affiliations:** 1 Department of Cellular and Molecular Biology, University of Texas Health Science Center at Tyler, Tyler, TX TX75708 USA; 2 Department of Medicine, National Jewish Health, Denver, CO USA; 3 Department of Biophysics, University of Texas Southwestern Medical Center, Dallas, TX USA; 4 Department of Biostatistics, Harvard T.H. Chan School of Public Health, Boston, MA USA; 5 Department of Biomedical Informatics, University of Pittsburgh School of Medicine, Pittsburgh, PA USA; 6 Division of Pulmonary Sciences and Critical Care Medicine, University of Colorado, Denver, CO USA

**Keywords:** Beryllium, Exposure assessment, Biomonitoring, Biomarker, Extracellular vesicles

## Abstract

**Purpose:**

Exposures related to beryllium (Be) are an enduring concern among workers in the nuclear weapons and other high-tech industries, calling for regular and rigorous biological monitoring. Conventional biomonitoring of Be in urine is not informative of cumulative exposure nor health outcomes. Biomarkers of exposure to Be based on non-invasive biomonitoring could help refine disease risk assessment. In a cohort of workers with Be exposure, we employed blood plasma extracellular vesicles (EVs) to discover novel biomarkers of exposure to Be.

**Methods:**

EVs were isolated from plasma using size-exclusion chromatography and subjected to mass spectrometry-based proteomics. A protein-based classifier was developed using LASSO regression and validated by ELISA.

**Results:**

We discovered a dual biomarker signature comprising zymogen granule protein 16B and putative protein FAM10A4 that differentiated between Be-exposed and -unexposed subjects. ELISA-based quantification of the biomarkers in an independent cohort of samples confirmed higher expression of the signature in the Be-exposed group, displaying high predictive accuracy (AUROC = 0.919). Furthermore, the biomarkers efficiently discriminated high- and low-exposure groups (AUROC = 0.749).

**Conclusions:**

This is the first report of EV biomarkers associated with Be exposure and exposure levels. The biomarkers could be implemented in resource-limited settings for Be exposure assessment.

**Supplementary Information:**

The online version contains supplementary material available at 10.1007/s00420-022-01871-7.

## Introduction

Beryllium (Be) is widely used in industry and occupational exposure to the low-density metal continues to be a serious concern due to its deleterious health effects (National-Research-Council 2008). Its physicochemical properties—such as being lighter in weight than other metals and having a unique combination of high thermal stability, heat-absorbing capacity, and electrical conductivity—make Be a valuable material in the aerospace, energy, defense, electronics, and automotive industries (National-Research-Council 2008). According to the US Department of Energy, emissions from coal-fired thermal power plants result in 100–1000 times higher Be concentration in the air compared to the typical ambient concentration (National-Research-Council 2008). Beryllium exposure can lead to beryllium sensitization (BeS), a cell-mediated immune response in susceptible individuals, which can progress to chronic beryllium disease (CBD), a granulomatous lung disease with no known cure (Occupational-Safety-and-Health-Administration 2017). Severe CBD can result in impaired quality of life, end-stage fibrotic lung disease, and death, especially in those with higher Be exposure. Exposure to Be is also associated with an increased risk of lung cancer, and the International Agency for Research on Cancer (IARC) lists Be as a group 1 carcinogen (Occupational-Safety-and-Health-Administration 2017). Very recently, the U.S. Occupational Safety and Health Administration (OSHA) revised the Be standard and reduced the Permissible Exposure Limit (PEL) for Be to 0.2 µg/m^3^ averaged over 8 h along with additional provisions such as medical screening and surveillance to protect workers exposed to Be. Unfortunately, despite the regulations, Be-induced lung disease continues to occur (Frye et al. 2021). The exposure–response relationship in sensitization and disease appears to be nonlinear; in some studies, higher exposures were associated with higher rates of CBD (Van Dyke et al. 2011a, b). Notably, BeS and CBD have been reported in subjects with exposure levels below the current National Institute for Occupational Safety and Health (NIOSH) Recommended Exposure Limit (REL) of 0.5 µg/m^3^ (https://www.cdc.gov/niosh/surveyreports/pdfs/263-13a.pdf). Biological monitoring of Be exposure based on measurement of Be levels in urine has no application for detecting exposure or any potential health effects (Apostoli and Schaller 2001; Centers-for-Disease-Control-and-Prevention 2017) as the metal concentrations in these biofluids do not reflect exposure associated with health outcomes nor any dose-response relationship. Specialized techniques such as X-ray fluorescence spectroscopy (XRF), which has been successfully used to assess concentration of metals such as lead in selective organs, are problematic for elements with atomic number less than 9 such as Be (Zawisza 2008). In situ detection of trace amounts of Be, that has atomic number 4, is not feasible using the state-of-the-art XRF. Novel biomarkers of Be exposure offers a promising alternative approach to estimate Be exposures and, more specifically, the internal dose. However, finding an effective biological matrix for non-invasive biomonitoring of Be exposure has remained a challenge.

Circulating proteins and metabolites (primary and secondary organic compounds) have been the most common types of biomolecules considered for use as biomarkers of exposure and disease. However, highly abundant blood proteins (~ 75 mg/ml) significantly interfere with protein biomarker discovery (Tu et al. 2010). Although immune-depletion strategies for high-abundance blood proteins have been proposed, they have been less efficient in discovery of low-abundance biomarkers in blood (Lee et al. 2019). Extracellular vesicles, involved in intercellular signaling, are a rich ‘multi-omic’ matrix comprising the transcriptome, proteome, lipidome, and metabolome. Most cell types in the body typically release around 6000–15,000 EVs per day (Riches et al. 2014; Agarwal et al. 2015; Chiu et al. 2016; Patel et al. 2017). We estimate that a single cell sheds about 1/1000th of its mass daily as EVs which could serve as surrogate nanobiopsy material reflecting the molecular and physiological status of the cell. Proteomic profiling of EVs is emerging as a promising approach for discovery of novel biomarkers and offers a threefold advantage: (1) proteomic analysis of EVs circumvents the need to deplete irrelevant high-abundance proteins that obscure the detection of low-abundance biomarker proteins; (2) the proteins enriched in circulating EVs from the blood are protected against degradation by the lipid bilayer membrane of the EVs; and (3) it is feasible to develop easy-to-adopt downstream quantitative assays for EV-based protein biomarkers such as Enzyme-linked Immunosorbent Assay (ELISA) or chemiluminescence-based assays for large-scale epidemiological studies without the need for high-end equipment. Thus, we posit that circulating EVs may enable researchers to overcome some of the key challenges in discovery of novel biomarkers for exposure assessments.

Findings from recent studies show that air pollutants (Sheller-Miller et al. 2020), cigarette smoke (Wu et al. 2019), alcohol (Crenshaw et al. 2019), obesity (Afrisham et al. 2020), nutrition (Maghraby et al. 2021), physical exercise (Fruhbeis et al. 2015), and oxidative stress (Chettimada et al. 2018) modify EV trafficking and alter EV composition. Monitoring exposure-associated effects (Harischandra et al. 2017) relying on EV biomarkers were recently reported. However, efforts toward discovery of biomarkers of exposure in EVs remain limited. In this study, we explore EVs as a biological matrix for discovery of biomarkers of exposure to Be. In a cohort of workers with history of occupational exposure to Be, we determined whether EV proteins could discriminate Be-exposed individuals from unexposed as well as distinguish between low and high Be exposure levels. We used mass spectrometry-based proteomic profiling of plasma EVs to discover biomarkers of Be exposure.

## Materials and methods

### Study participants

Plasma samples of Be-exposed subjects were collected between the years 2001 and 2014 as part of the Beryllium BioBank (BBB), a multi-center Department of Energy funded repository to study beryllium-related disease, and deposited into the chronic beryllium biorepository at National Jewish Health (NJH), Denver, Colorado (Newman et al. 2012). This study was approved by institutional ethics committee of NJH (#HS 2466) and University of Texas Health Science Center at Tyler (IRB #20–019 & #0,000,370). Plasma of healthy age-matched unexposed subjects was obtained from biorepository of Discovery Life Sciences, Huntsville, Alabama. The study was conducted on a total of 143 subjects including 31 healthy subjects not exposed to Be (hereafter referred as ‘unexposed’) and 112 subjects exposed to Be (hereafter referred as ‘exposed’). The set of exposed subjects consisted of 36 healthy exposed subjects without beryllium health effects (HE), 38 BeS, and 38 CBD. The 31 unexposed subjects selected for the study belonged to general population not engaged in occupations or industries involving Be compounds. Subjects were divided into discovery and validation cohorts. For each cohort, exposed subjects were randomly selected, and then unexposed subjects matching demographic variables such as age and gender were manually selected. A total of 64 subjects consisting of 48 exposed and 16 unexposed were considered as discovery cohort. On this cohort, the protein signature was identified using mass spectrometry-based proteomic profiling. The remaining 64 exposed and 15 unexposed subjects were considered for biomarker validation (validation cohort) by ELISA. Peripheral blood was previously collected from participants in EDTA tubes after informed consent. Plasma was separated from blood no later than 1 h from the time of collection by centrifugation at 1100×*g* for 10 min at room temperature and stored at − 80 °C. Age, gender, and ethnicity were collected from the BBB. Lifetime cumulative exposures were determined using methods previously described (Newman et al. 2012; Crooks et al. 2021) and expressed as µg/m^3^. The work histories of Be-exposed subjects were collected using an interviewer-administered questionnaire and company work history records. To ensure that uniform data were obtained from all subjects, BBB staff provided training to those administering the questionnaires. This included training from an Industrial Hygienist in the administration of the exposure questionnaire. Information collected included facility, hire and termination dates, job titles with dates held, and longest job held. The longest held job title was categorized into one of 14 job categories as follows: machinist; IH/safety; administration; custodial/maintenance; construction worker; in plant trades person; engineer/research and development; inspector/quality control; industrial production (non-machinist); non-production in plant; decontamination and decommissioning (D&D); laboratory; security; and management. Past and current industrial hygiene data and information, historical industry data, published data, and data collected for regulatory purposes were used to calculate facility specific exposure estimates for each of the 14 job categories. An arithmetic mean of the available exposure measurements was calculated for each category within time periods based on years of data collection. Cumulative exposure estimates for each participant were calculated by multiplying the work-time in each job category by corresponding time period exposure estimate.

### Preparation of extracellular vesicle samples

Plasma was clarified by serial centrifugations at 2500*g*, 10,000*g*, and 30,000*g*, each for 15 min at 4 °C. EVs were isolated using size-exclusion chromatography with qEV original 35 nm pore size columns and automated fraction collector V1 setup (Izon Science US Ltd, MA, USA), as per manufacturer’s recommendation. Briefly, columns were equilibrated with degassed 1X phosphate-buffered saline (PBS) at pH 7.4 and 500 µL of clarified plasma was allowed to enter the column by gravity flow. The buffer reservoir was filled with fresh PBS and 3 mL of void volume and four EV fractions of 500 µL each were collected. The fractions were pooled together and concentrated using 300 KDa centrifugal filters (Pall corp, NY, USA) at 3500 g at 4 °C and stored in aliquots at − 80 °C prior to analysis.

### Cryo-electron microscopy of extracellular vesicles

Three to four microliters of the EV solution (1:20 dilution) was added to Lacey carbon grids (300-mesh; Ted Pella, Inc., Redding, CA, USA) that were negatively glow-discharged for 80 s at 30 mA. Excess sample was removed by blotting once for 3 s with Vitrobot filter paper (Ted Pella, Inc, Redding, CA, USA) and then the grid was plunge-frozen in liquid ethane cooled by liquid nitrogen using a Vitrobot plunge-freezer (ThermoFisher Scientific, Hillsboro, OR, USA).

The vitrified vesicle samples were imaged using a Talos Arctica 200 kV transmission electron microscope (ThermoFisher Scientific, Hillsboro, OR, USA) equipped with a Gatan K3 camera (Gatan, Inc., Pleasanton, CA, USA) The SerialEM software was used to collect images under low-dose conditions at 36,000 × magnification corresponding to a pixel size of 1.14 Å/pixel. For each image, 50 frames were recorded over 2.5 s exposure time at a dose rate of 35 electrons/pixel/s. The movie frames were aligned using MotionCorr2 (2) under Relion (Zivanov et al. 2018).

### Nanoparticle tracking analysis (NTA) of extracellular vesicles

Concentration and size range of plasma EV preparations were measured using Zetaview Quatt instrument (Particle Metrix, Germany) in scatter mode using 520 nm laser and sCMOS camera. Instrument calibration was performed with 100 nm fluorescent polystyrene beads. PBS filtered through 0.2 μm syringe filter was used for diluting the EVs to appropriate concentration. Data were acquired at the following settings for each channel: sensitivity = 80; shutter = 100; track length = 15; minimum brightness = 20; cycles/position = 2/11.

### Lysis of extracellular vesicles protein estimation

The EV fractions from SEC were concentrated down to 50 µL in vacuum at 30 °C, lysed in RIPA buffer (Sigma Aldrich, USA), incubated on ice for 10 min and at 95 °C for 10 min, and sonicated for 2 min in an ultrasonic bath sonicator (Branson Ultrasonics Corp, CT, USA). The supernatants containing the EV proteins were collected after centrifugation at 30,000*g* for 15 min at 4 °C. The protein concentration was estimated using a Pierce BCA protein assay kit (Thermo Fisher Scientific, MA, USA) using bovine serum albumin as standard.

### Western blot

Western blots were performed following SDS PAGE using standard protocols involving anti-CD81 antibody (Cat# ab232390) from Abcam, Waltham, MA, USA; anti-TSG101 antibody (Cat# SC-7964), anti-HSP70 antibody (Cat# SC-24) and anti-Calnexin antibody (Cat# SC-23954) from Santa Cruz Biotechnology, CA, USA. Following incubation with appropriate secondary antibody, proteins were detected by chemiluminescence using Clarity Max ECL substrate (Bio-Rad Laboratories, Hercules, California, USA) on a Chemidoc-MP Gel imaging system (Bio-Rad Laboratories, Hercules, California, USA).

### Liquid chromatography–mass spectrometry/mass spectrometry analysis

Samples were digested overnight with trypsin (Pierce, Rockford, IL, USA) following reduction and alkylation with DTT and iodoacetamide (Sigma–Aldrich, St. Louis, MO, USA). The samples then underwent solid-phase extraction cleanup with an Oasis HLB plate (Waters, Milford, MA, USA) and were subsequently dried and reconstituted in 10 µL of 2% ACN, 0.1% TFA. 5 µL of these samples was injected onto a QExactive HF mass spectrometer coupled to an Ultimate 3000 RSLC-Nano liquid chromatography system. Samples were injected onto a 75 µm i.d., 15-cm-long EasySpray column (Thermo Scientific, San Jose, CA, USA) and eluted with a gradient from 0 to 28% buffer B over 90 min with a flow rate of 250 nL/min. Buffer A contained 2% (v/v) ACN and 0.1% formic acid in water, and buffer B contained 80% (v/v) ACN, 10% (v/v) trifluoroethanol, and 0.1% formic acid in water. The mass spectrometer operated in positive ion mode with a source voltage of 2.2 kV and an ion transfer tube temperature of 275 °C. MS scans were acquired at 120,000 resolution in the Orbitrap and up to 20 MS/MS spectra were obtained for each full spectrum acquired using higher energy collisional dissociation (HCD) for ions with charges 2–8. Dynamic exclusion was set for 20 s after an ion was selected for fragmentation.

Raw MS data files were analyzed using Proteome Discoverer v2.4 (Thermo Scientific, San Jose, CA, USA), with peptide identification performed using Sequest HT searching against the human protein database from UniProt (downloaded on March 12, 2020). Fragment and precursor tolerances of 10 ppm and 0.02 Da were specified, and three missed cleavages were allowed. Carbamidomethylation of Cys was set as a fixed modification, with oxidation of Met set as a variable modification. The false-discovery rate (FDR) cutoff was 1% for all peptides.

### Enzyme-linked immunosorbent assay (ELISA)

Standard sandwich ELISA was performed for ZG16B (Aviva Systems Biology, CA, USA) and ST13P4 (Abbexa LLC, TX, USA) as per manufacturer’s instructions and the microplates were read at 450 nm on a Synergy microplate reader (BioTek Instruments Ltd, VT, USA).

### Biomarker discovery and validation

The biomarker panel was developed using mass spectrometry-based protein quantification of plasma EV proteins. For each protein, unpaired *t* tests were performed on the log(n + 1) transformed abundance values to identify differentially expressed EV proteins in different classes (exposed vs. unexposed). The Benjamini–Hochberg (BH) multiple testing correction was used to control FDR in differential expression analysis. Genes exhibiting |log2FC|> 0.585 (equivalent to > 50% increase or decrease in expression) and BH-adjusted *p* value < 0.05 were considered differentially expressed (DE) (log2FC = log_2_ fold change). The upregulated DE proteins were subjected to least absolute shrinkage and selection operator (LASSO) regression to identify a biomarker panel discriminating two different classes of samples. The LASSO tuning parameter (λ) was optimized using tenfold cross-validation. We chose the largest value of λ for which the cross-validated error is within one standard error of the minimum cross-validation error; this value is denoted λ.1se. The receiver-operating characteristic (ROC) curve and the area under the ROC curve (AUROC) were computed to evaluate the performance of the classifier.

The biomarker panel identified in discovery cohort using LASSO regression was validated by ELISA in an independent set of samples. For each protein, unpaired t-tests were used to detect significant differences in the expression between (a) exposed versus unexposed subjects and (b) low-exposure versus high-exposure subjects. In addition, a classifier was constructed by fitting a logistic regression model on the protein concentrations obtained via ELISA. The performance of the classifier was evaluated using ROC curves and by calculating AUROC values (Hajian-Tilaki 2013). The logistic regression classifier was further evaluated by plotting calibration curves and calculating the concordance index (Frank and Harrell 2015). Leave-one-out cross-validation using caret package was used to further evaluate the performance of the classifier. Nomograms were constructed for binary classification to facilitate easy interpretation of the logistic regression model. All statistical analyses were performed in the R environment (R4.0.3).

## Results

### Study subjects

Demographic and clinical features of the subjects participating in the study are shown in Tables 1, 2. The average age of the study subjects was 64.13 years with a range of 33–92 years. The cumulative exposure ranged between 0.00025 and 62.09 µg/m^3^ with a mean and median of 4.45 and 0.806 µg/m^3^, respectively. The study population was predominantly male (81.1%) and included former or current smokers (57.3%). The study population was divided into two cohorts, namely the discovery cohort (Table 1) and the validation cohort (Table 2). The discovery cohort consisted of 64 subjects, of which 48 were exposed to Be and 16 were unexposed. Among the 48 exposed subjects, there were equal numbers of subjects belonging to the healthy (HE), BeS, and CBD categories. The validation cohort comprised 15 unexposed subjects and 64 exposed subjects including 22, 20, and 22 subjects belonging to HE, BeS, and CBD categories, respectively.

**Table 1:** Demographic and exposure characteristics of workers in discovery cohort

	Unexposed	Exposed		
		HE	BeS	CBD
Total (N)	16	16	16	16
Gender (*n*)	14	13	16	12
Male	2	3	0	4
Female				
Age	62.3 (33–80)	69.4 (54–84)	57.4 (43–73)	64.6 (43–82)
Smoking (*n*)				
Never	4	5	7	10
Former	3	11	6	6
Current	9	0	3	0
Cumulative exposure (µg/m^3^)		4.74 (0.00375–25.2)	5.49 (0.00375–41.51)	5.96 (0.026–51.59)
Cumulative exposure (*n*)				
Low		3	7	4
Intermediate		5	1	4
High		8	8	8

**Table 2:** Demographic and exposure characteristics of workers in validation cohort

	Unexposed	Exposed		
		HE	BeS	CBD
Total (N)	15	22	20	22
Gender (*n*)				
Male	13	15	14	19
Female	2	7	6	3
Age	65.1 (45–70)	70.7 (54–85)	59.1 (43–83)	63.5 (43–92)
Smoking (*n*)				
Never	4	7	13	10
Former	3	12	6	12
Current	7	3	1	0
NA	1	0	0	0
Cumulative exposure (µg/m^3^)		2.07 (0.04–10.24)	1.71 (0.014–17.82)	7.26 (0.00025–62.09)
Cumulative exposure				
Low		7	7	7
Intermediate		8	5	4
High		7	8	11

### EV characteristics

**Fig. 1:**
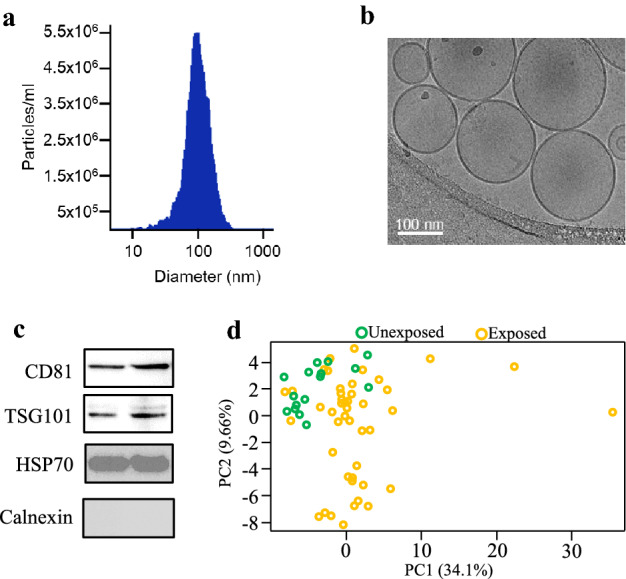
EV characterization and principal components analysis of EV proteomes. **a** Size and quantification of plasma EVs measured by nanoparticle tracking analysis against 0.1 μm fluorescent polystyrene beads standard. Graph shows EV concentration after 3000 times dilution. **b** Representative cryo-electron micrograph of EVs isolated from peripheral blood plasma of participants in the study. **c** Detection of common EV markers using Western blot for two representative plasma samples. **d** Principal components analysis of proteomic profiles of control and exposed subjects

EVs isolated from plasma using size-exclusion chromatography exhibited an average diameter of 110 nm on nanoparticle tracking analysis (Fig. 1a). Plasma contained around 2 × 10^10^ EVs per mL (Fig. 1a). The EVs were seen in spherical shape, possessed a lipid bilayer on cryo-electron microscopic examination (Fig. 1b), and were predominantly of ~ 100 nm in diameter, further confirming results obtained in NTA. In addition, the EV preparations contained the tetraspanin transmembrane protein like CD81, and exosome luminal protein such as TSG101 and HSP70, while they were devoid of Calnexin, an endoplasmic reticulum membrane protein (Fig. 1c). Altogether, these results suggest that our EV preparations were predominantly enriched with exosomes.

### EV-based biomarker discovery for discriminating Be-exposed subjects from unexposed subjects

**Fig. 2:**
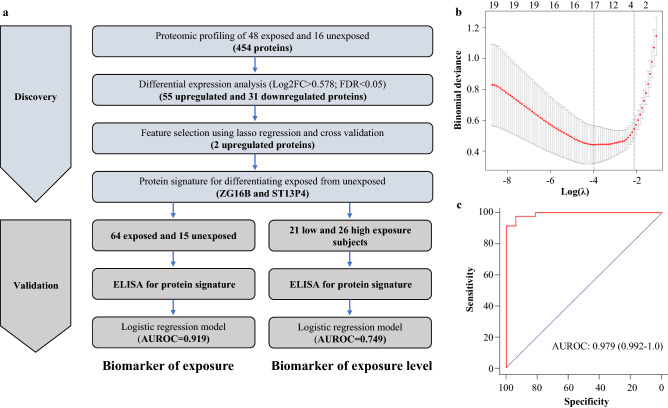
Discovery of EV protein biomarker signature for discriminating exposed and unexposed subjects. **a** Flow chart depicting the study design of systematic discovery of EV protein biomarker signature. **b** Plot showing cross-validation error at different values of tuning parameter (λ) in LASSO regression in cohort-I. Red dotted curve shows the tenfold cross-validation curve while the whiskers represent ± one standard deviation, respectively. The vertical dotted lines show the locations of λ.min and λ.1SE. The numbers across the top are the number of nonzero coefficients obtained when using each given value of λ. **c** ROC curve for logistic regression model employing ZG16B and ST13P, evaluated on the discovery cohort

Mass spectrometry-based proteomic analysis of plasma EVs from the discovery cohort quantified the expression of 454 proteins across 64 samples. Dimension reduction using principal components analysis revealed that plasma EVs from exposed subjects exhibited distinct proteomic profiles compared to unexposed subjects (Fig. 1d). A schematic representation of our biomarker discovery process is depicted in (Fig. 2a). Differential expression analysis revealed that there were 55 upregulated proteins and 31 downregulated proteins in exposed subjects compared to controls, as determined by the set of proteins with BH-adjusted *p* value < 0.05 and |log2FC|> 0.585 (Table S1). Since upregulated proteins tend to be more suitable as biomarkers for diagnostic use in clinical settings, we selected upregulated proteins for further analysis. We used LASSO regression on the 55 upregulated proteins to identify a signature that discriminates exposed individuals from unexposed subjects. The 1SE rule for choosing the LASSO tuning parameter λ, as described above, yielded λ = − 2.1, which produced a classifier employing two proteins, namely zymogen granule protein 16B (ZG16B) and putative protein (ST13P4), for discriminating exposed from unexposed subjects (Fig. 2b). To assess the efficacy of the two-protein biomarker panel that was identified in the discovery cohort (ZG16B and ST13P4), we evaluated the performance of a logistic regression model using these two proteins on the discovery cohort. ROC curves based on the logistic regression model yielded an AUROC of 0.992 (95% CI: 0.979–1.0) suggesting that the biomarker signature has excellent efficiency in identification of exposed subjects (Fig. 2c).

### Validation of EV protein signature by ELISA in an independent cohort

**Fig. 3:**
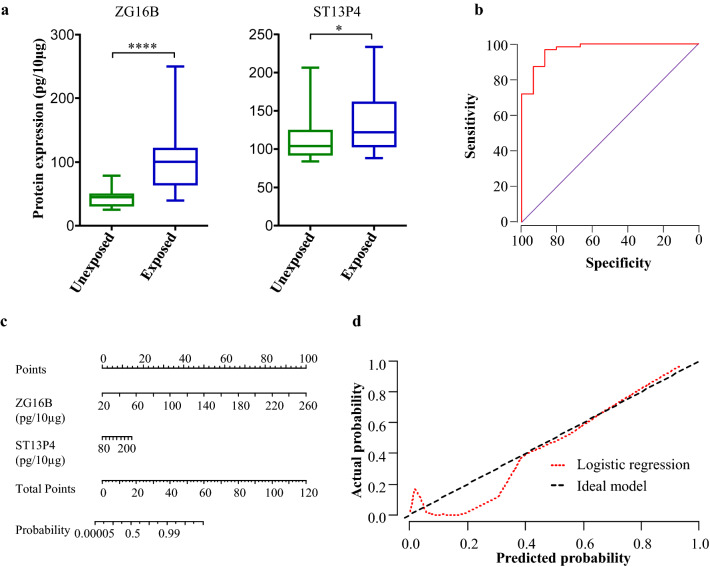
Independent validation of two-protein signature for discriminating exposed and unexposed subjects. **a**) Expression of two-protein signature in plasma EVs of exposed and unexposed subjects in validation cohort quantified using ELISA. **b** Receiver-operating characteristic (ROC) curve, **c** nomogram, and **d** calibration curve for the logistic regression model, evaluated on the validation cohort

To evaluate the clinical utility of the two-protein signature, in an independent cohort of samples (validation cohort), we measured the expression levels of these proteins in plasma EVs using ELISA. The proteins ZG16B and ST13P4 were 2.1 (*p* = 0.0001) and 1.5 (*p* = 0.048)-fold higher, respectively, in exposed subjects compared to unexposed (Fig. 3a). We further examined whether the biomarker levels were influenced by disease states. The expression of these two proteins was not significantly different between HE, BeS and CBD groups (Figure S1A). To evaluate the performance of these two proteins for discriminating between exposed and unexposed subjects, we performed logistic regression and constructed ROC curves (Fig. 3b). The protein signature exhibited an excellent AUROC value of 0.919, with 95% sensitivity at 98% specificity. We performed leave-one-out cross-validation to further test the performance of the signature to discriminate exposed and unexposed subjects. Out of 79 cohort samples analyzed in the logistic regression model, 70 (88.6%) were correctly classified in cross-validation while two unexposed and seven exposed samples were misclassified. For easier graphical interpretation of the logistic regression model, we constructed a nomogram for the two-protein classifier (Fig. 3c). To assess the calibration of logistic regression model, we plotted calibration plot showing predicted and actual probabilities on *X* and *Y* axis, respectively (Fig. 3d). Our model showed excellent consistency in discriminating between the two groups. Furthermore, the concordance index of the model was calculated using 200 bootstrap samples to evaluate the predictive accuracy. Our logistic regression model obtained a concordance index value of 0.86 (95% CI: 0.81–0.93) suggesting that it differentiates exposed and unexposed subjects with high accuracy.

### Two-protein signature could be useful in differentiating between subjects with high and low levels of cumulative Be exposure

**Fig. 4:**
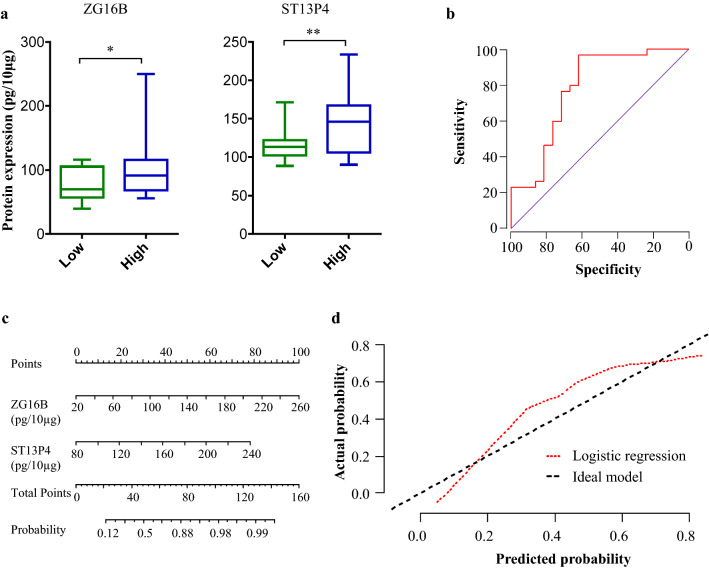
Validation of the two-protein signature for discriminating subjects with high and low cumulative exposure. **a** Expression of two-protein signature in plasma EVs of low- and high-exposure groups in validation cohort quantified using ELISA. **b** Receiver-operating characteristic (AUROC) curve, **c**) nomogram, and **d** calibration curve for the logistic regression model, evaluated on the validation cohort

Encouraged by the predictive accuracy of the two-protein biomarker signature of exposure to Be in an independent validation cohort, we next investigated whether the same protein signature could also differentiate subjects with high-exposure level from low-exposure level. Subjects with cumulative exposure levels below 0.25 µg/m^3^ and above 1.0 µg/m^3^ were classified as having low and high exposure, respectively. Subjects with intermediate cumulative exposure levels between 0.25 µg/m^3^ and 1.0 µg/m^3^ were excluded from this analysis since we anticipated the protein expression patterns in this intermediate group to overlap with the high or low-exposure groups. There were 21 and 26 subjects with low- and high-exposure level, respectively, in the validation cohort. The levels of ZG16B and ST13P4 were 1.7 (*p* = 0.05) and 1.8 (*p* = 0.004)-fold higher, respectively, in high-exposure subjects compared to low-exposure (Fig. 4a). Logistic regression models followed by ROC curves were constructed to evaluate the performance of these two proteins for discriminating between high- and low-exposure groups (Fig. 4b). The protein signature showed an AUROC value of 0.749, with 81% sensitivity at 80% specificity. We performed leave-one-out cross-validation to further test the performance of the signature in discriminating low- and high-exposure levels. Among the 47 samples analyzed in the logistic regression model, 32 samples (68.1%) were correctly classified in cross-validation while seven samples in low-exposure category and eight in high-exposure category were misclassified. Further, we constructed a nomogram for this logistic regression model comprising the two proteins (Fig. 4c) and plotted the calibration curve (Fig. 4d). The biomarker signature showed good consistency in discrimination of high versus low exposure. Furthermore, the model obtained a concordance index of 0.68 (95% CI: 0.59–0.78) after 200 bootstrap samples, suggesting that the two-protein signature had a good predictive power in discriminating exposure levels.

## Discussion

Due to new industrial applications of beryllium compounds, occupational exposure to Be is likely to continue (National-Research-Council 2008). There is considerable dispute about which factors increase the risk for developing Be-related illnesses. There has been considerable interest in understanding the genetic factors that predispose an individual to develop BeS or CBD and few disease-specific genetic markers have been studied previously (Richeldi et al. 1993; Richeldi et al. 1997; Maier et al. 1999; Wang et al. 1999; Maier et al. 2001; Saltini et al. 2001; Wang et al. 2001; Maier et al. 2002; Rossman et al. 2002; Maier et al. 2003; McCanlies et al. 2004). Studies have demonstrated that a linear dose–response does not appear to occur in the case of Be, since exposure to very low levels of Be has been reported to cause BeS and CBD in some individuals (National-Research-Council 2008). Further, while overall, there are clear genetic and environmental factors involved in susceptibility to Be-related illnesses, the specifics as well as the ability to predict risk for individual workers are not well defined. Therefore, early detection of Be exposure could improve clinical management of Be-related health hazards and may help prevent or delay disease onset.

Historically, assessment of exposure to Be has relied on direct measurements by personal monitoring in occupational microenvironments and indirect estimates based on time-activity information from questionnaires and interviews (Occupational-Safety-and-Health-Administration 2017). Although monitoring Be exposure based on personal breathing zone air samples that reflect the airborne exposure may provide short-term exposure measurements, extrapolation of the short-term exposure data to lifetime exposure measurements for risk assessment purposes is not straight forward. Furthermore, exposure is only a proxy for quantifying the internal dose, which is the amount of Be deposited within the body. Thus, although personal monitoring over longer periods of time and at regular intervals may provide a better exposure assessment, in reality, this is not feasible for workplaces and significantly different internal doses may result from the same exposure. Further, biomonitoring by directly measuring Be levels in accessible biological fluids do not reflect cumulative exposure or internal dose (Apostoli and Schaller 2001). Previously, changes in the serum cytokine levels of TNFα and IL-6 along with their respective soluble receptor levels have been investigated from the context of differential diagnosis of Be-related disease (Tinkle and Newman 1997). However, to date, no blood- or urine-based biomarkers of exposure to Be that reflect lifetime work exposure have been reported. Thus, discovery and development of novel biomarkers that accurately reflect lifetime cumulative exposure with a potential step towards risk assessment could be very valuable.

Leveraging an extensive repository of plasma samples from a well-characterized cohort of workers with history of occupational exposure to Be, we discovered and developed a robust biomarker panel that can be analyzed by ELISA to determine whether exposure to Be had occurred as well as predict a relative internal dose. This assay does not require sophisticated laboratory infrastructure and can be performed in a clinical laboratory. We show that EVs provide a biomarker signature with excellent power for differentiating Be-exposed individuals from unexposed. We acknowledge that the biomarker panel misclassified a few individuals mostly in the low-exposure category as being unexposed. Since the archived plasma samples used in this study were collected years after cessation of Be exposure, it is possible that the imperfect classification performance on low-exposure individuals could be attributed to the Be kinetics and related variables affecting internal dose. Currently, specific information related to the exact composition of the Be mixture, internal doses in the exposed cohort, and general information on organ-wise clearance kinetics of Be in humans is unknown or undetermined.

Interestingly, we found that the same two-protein signature could distinguish between low and high cumulative exposure groups. Our biomarker evaluations show that the classifier can stratify individuals based on the exposure level and it exhibited an exposure–biomarker response relationship. Since cumulative exposure is known to be a good predictor of CBD risk, improved estimates of cumulative exposure may help in risk stratification of workers. Further, since biomarkers are likely to be more informative of internal dose than exposure monitoring, it is possible that the biomarker signature may be even more effective than these performance evaluations would suggest. We envisage future use of physiologically based pharmacokinetic/pharmacodynamic (PBPK/PD) models in conjunction with the predictive power of the EV biomarkers for making accurate measurements of Be internal dose.

Our study had some limitations inherent to retrospective studies characterizing the exposure–biomarker relationship. First, a common limitation of job-exposure matrix-based retrospective exposure assessment is that the time-activity profiles (time spent in various jobs) based on which lifetime cumulative exposures are estimated, in many instances, does not account for spatial and temporal variability in ambient metal concentrations. Therefore, the lifetime cumulative exposure assessment may not reflect true total exposure. Second, to delineate the exposure–biomarker relationship, we made the fundamental assumption that ambient exposure metal concentrations are a surrogate for personal exposure as well as internal dose. However, the amount of internal dose would depend on several factors such as inhaled concentration, ventilation rate, and fractional deposition. Furthermore, due to inadequate data on the biokinetics and bioavailability of Be in humans, our study lacked sufficient information to determine the internal dose. For these reasons, this biomarker panel should be validated in large prospective cohorts to establish the reproducibility of biomarkers. Lastly, this study was conducted on workers in nuclear weapons facility exposed to pure form of Be. Therefore, these biomarkers need to be validated in occupational settings involving other Be compounds. Despite these limitations, the biomarker panel described in this study may help in developing a screening test for detecting exposure to pure Be and potentially identify workers in the US with a high propensity for developing CBD (Van Dyke et al. 2011a, b).

## Conclusions

Biological monitoring is a valuable approach to assess the potential health risk from exposure to hazardous agents. Lack of reliable biomarkers is a major challenge facing biological monitoring of Be, which otherwise could aid in monitoring health status of individuals exposed to Be at workplace. The direct measurement of Be in biofluids such as urine or blood does not reflect the total body burden. Our study demonstrates the feasibility of mass spectrometry analysis of EVs to discover novel protein biomarkers for identifying Be-exposed individuals and stratifying them based on the exposure levels. Expansion of this proof-of-concept biomarker paradigm could provide a framework for measuring cumulative exposures of other hazardous agents posing threats to human health.

## Supplementary Information

Below is the link to the electronic supplementary material.

Supplementary file1 (PDF 132 KB)

## Data Availability

All the relevant data were included in the manuscript and the supplementary document.
