# Supplementary material for: Realistic biomarkers from plasma extracellular vesicles for detection of beryllium exposure
Source: Int Arch Occup Environ Health. Author manuscript; Available in PMC 2022 Oct 1. (PMC9489591; doi:10.1007/s00420-022-01871-7)
Supplement: Supplementray file [file NIHMS1809400-supplement-Supplementray_file.pdf]

Supplementary information

Plasma Extracellular Vesicles Protein Signature for Detection of Beryllium Exposure

Raju SR. Adduri[1], Ravikiran Vasireddy[1], Margaret M. Mroz[2], Anisha Bhakta[1], Yang Li[3], Zhe Chen[3], Jeffrey W. Miller[4], Karen Y Velasco-Alzate[1], Vanathi Gopalakrishnan[5], Lisa A. Maier[2,6], Li Li[2,6], Nagarjun V. Konduru[1*]

[1]Department of Cellular and Molecular Biology, University of Texas Health Science Center at Tyler, Tyler, Texas, USA.

[2]Department of Medicine, National Jewish Health, Denver, Colorado, USA.

[3]Department of Biophysics, University of Texas Southwestern Medical Center, Dallas, Texas, USA.

[4]Department of Biostatistics, Harvard T.H. Chan School of Public Health, Boston, Massachusetts, USA.

[5]Department of Biomedical Informatics, University of Pittsburgh School of Medicine, Pittsburgh, Pennsylvania, USA.

[6]Division of Pulmonary Sciences and Critical Care Medicine, University of Colorado, Denver, Colorado, USA.

***Corresponding author:** Nagarjun V Konduru, Assistant Professor, Department of Cellular and Molecular Biology, University of Texas Health Science Center at Tyler, Tyler, TX75708, USA. Email: Nagarjun.KonduruVenkata@uthct.edu

Table S1. Differentially expressed genes in beryllium exposed workers compared to unexposed subjects

Protein	p value	BH	Log2FC
CTSG	8.4E-08	6.27E-06	-11.5441
COLEC11	0.000022	0.000476	-10.8719
PTPRJ	0.000729	0.007767	-9.98917
AGBL3	0.000913	0.009216	-9.81842
FBN1	9.25E-05	0.00175	-8.25454
ADAM10	0.004553	0.031798	-7.94368
FLG	0.000248	0.003631	-7.55242
FYB; FYB1	0.000392	0.005083	-7.43147
FAT4	0.005223	0.033521	-7.11142
GGH	0.000445	0.005457	-6.6525
LAMP2	0.000439	0.005457	-5.43934
HRNR	0.00872	0.047659	-5.34372
KNG1	0.00135	0.012019	-4.80971
HEG1	0.004893	0.033521	-4.76613
LAP3	0.000569	0.006625	-4.49113
LOR	0.000654	0.007251	-4.13834
MUC5AC	0.002066	0.01646	-4.07971
KATNAL1	0.00499	0.033521	-3.55813
LAMB2	0.008923	0.047659	-2.96947
LTBP1	0.007722	0.043081	-2.70198
LRRC15	0.008886	0.047659	-2.54057
MUC16	0.00183	0.015382	-2.13682
VAMP3	3.03E-08	3.54E-06	-2.08908
PTGDS	2.46E-07	1.4E-05	-2.07908
YWHAZ	4.78E-06	0.000167	-1.70449
SMIM5	0.009078	0.047923	-1.22792
PLEC	0.000179	0.002861	-1.18044
REG3A	8.02E-06	0.000228	-1.06161
PKHD1L1	0.000503	0.006006	-0.93193
VCP	0.006746	0.039473	-0.73188
RPLP0	0.005179	0.033521	-0.64218
ZNF525	0.00439	0.031142	0.652092
SLC2A3	0.005048	0.033521	0.709736
TFRC	0.007089	0.040231	0.725466
SERPINA1	0.000183	0.002861	0.889372
SDR9C7	0.005242	0.033521	0.917289
PLD3	3.52E-08	3.54E-06	0.944808
PTRF; CAVIN1	0.001086	0.010494	0.993917

S100A16	0.001384	0.012086	1.014288
STX7	0.005426	0.034215	1.119711
SEPP1; SELENOP	0.002843	0.021509	1.13773
RAB27B	0.007781	0.043081	1.188232
PIP	0.002067	0.01646	1.376261
VCAN	0.001255	0.01163	1.433676
SERPIND1	0.000275	0.003781	1.441918
ST13P4	1.36E-09	3.09E-07	1.586215
SLC2A1	0.000128	0.00227	1.717787
SPIN4	0.001585	0.013578	1.742336
PRDX2	4.41E-07	2.22E-05	1.747592
SPARCL1	0.000219	0.003311	1.748128
SSFA2	1.14E-05	0.000288	1.871406
SSC5D	3.61E-06	0.000137	1.948387
ZG16B	2.17E-13	9.85E-11	2.037513
SELL	0.000857	0.008838	2.050825
S100A4	1.85E-05	0.00042	2.344032
KIAA0825	0.006839	0.039473	2.486868
KIAA2012	0.006869	0.039473	2.618066
MYCT1	0.001905	0.015722	3.278532
PZP	0.00035	0.004676	3.473196
ANGPTL6	0.005692	0.035286	4.495806
SPRR3	0.00013	0.00227	4.734145
FN1	6.57E-05	0.001356	5.065197
SDC1	0.00026	0.003696	5.122864
ARG1	0.001334	0.012019	5.43662
ANXA7	6.82E-06	0.00022	6.213825
MMRN1	0.003953	0.028487	6.287519
ANXA11	7.28E-06	0.00022	6.461624
ALAD	1.48E-05	0.000354	6.517663
COL6A3	0.00211	0.016519	6.754763
AZGP1	1.33E-06	5.49E-05	6.803184
CELSR2	0.000655	0.007251	6.841304
CAT	8.97E-05	0.00175	7.074747
C1orf68	6.03E-07	2.74E-05	7.384421
HMGB1	0.009397	0.049037	7.404713
DSG1	0.003848	0.028176	7.455191
GTF3A	0.006389	0.038166	7.508493
EXTL2	0.002505	0.019275	7.792809
CCDC73	3.9E-08	3.54E-06	8.283244
FETUB	0.00598	0.036201	8.430444

CASP14	9.67E-08	6.27E-06	8.621735
CEACAM6	0.00001	0.000267	8.650556
GNPTG	0.00094	0.009279	9.226178
DSC3	0.000166	0.002789	9.594199
IGFALS	0.000736	0.007767	9.790398
ICAM1	0.00113	0.010687	10.14826

BH, Benjamini-Hochberg corrected p value; Log2FC, Log2 fold change

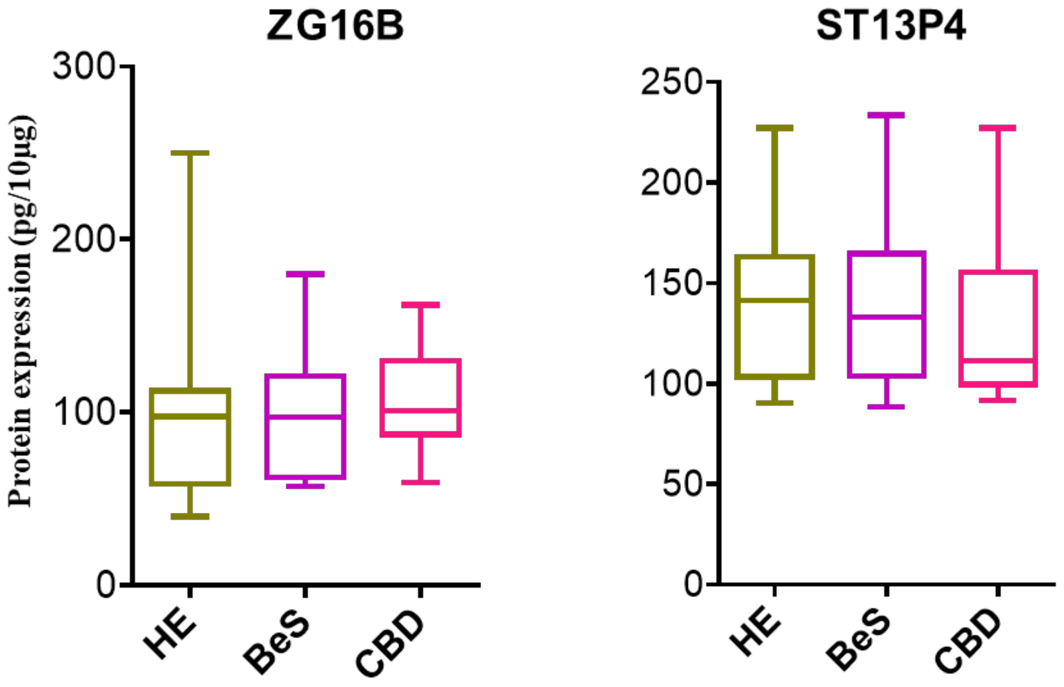

Figure S1. Expression of the two protein signature in plasma EVs of HE, BeS and CBD groups in validation cohort.
